# Mycobacterium tuberculosis Infection in the Setting of Interstitial Lung Disease: Coincidence or Bad Luck?

**DOI:** 10.7759/cureus.3391

**Published:** 2018-10-01

**Authors:** Christian Wong, Sonu Sahni, Muhammad Azaz I Cheema, Asma Iftikhar

**Affiliations:** 1 Internal Medicine, Touro College of Osteopathic Medicine, New York, USA; 2 Internal Medicine, Brookdale University Hospital Medical Center, New York, USA; 3 Pulmonology, New York Presbyterian Hospital Queens, New York, USA

**Keywords:** interstitial lung disease, mycobacterium, tuberculosis, infectious disease, pulmonary diseases, radiology

## Abstract

Mycobacterium tuberculosis (MTB) infection is the ninth leading cause of death worldwide, with many individuals with undiagnosed active or latent disease. The presence of parenchymal lung disease, such as interstitial lung disease (ILD), has been suggested to increase the risk of pulmonary tuberculosis (TB). In the clinical setting of ILD, the diagnosis of an underlying MTB infection may be challenging due to the interstitial process and underlying fibrosis, which may mask the infection. An atypical presentation and misleading radiological patterns may delay the diagnosis of the underlying MTB infection. Herein, we describe a unique case of ILD complicated by active MTB infection in an 84-year-old male, which presented as a diagnostic and clinical challenge. Eventually, due to hypoxia and respiratory failure, the patient expired.

## Introduction

Mycobacterium tuberculosis (MTB) infection is the ninth leading cause of death worldwide and the leading cause from a single infectious agent, ranking above human immunodeficiency virus infection/acquired immune deficiency syndrome (HIV/AIDS) [1]. With approximately one-third of the world’s population having been exposed to MTB, there exist many individuals with undiagnosed active or latent disease [1]. Susceptibility to infection may be due to a compromise of the immune system, the use of immune-modulating drugs, or an underlying disease such as bronchogenic cancer [2]. It has also been described in the literature that the presence of parenchymal lung disease, such as idiopathic pulmonary fibrosis (IPF), a type of interstitial lung disease (ILD), increases the risk of pulmonary tuberculosis (TB) [3].

ILD is an umbrella term for a group of disorders that present with a similar constellation of signs and symptoms, including progressively worsening dyspnea on exertion, dry cough, and bibasilar inspiratory crackles on auscultation. Aside from IPF, other causes of ILD include occupational work exposures, fungal etiology, or atypical bacterial pneumonia. In the clinical setting of ILD, the diagnosis of an underlying MTB infection may be challenging due to the interstitial process and underlying fibrosis, which may mask the infection. Herein, we describe a unique case of ILD complicated by an active MTB infection in an 84-year-old male, which presented as a diagnostic and clinical challenge.

## Case presentation

An 84-year-old, Ecuadorian, non-smoker, Spanish-speaking male with a past medical history of dementia, hypertension, and hyperparathyroidism presented with cough productive of purulent sputum associated with persistent shortness of breath and non-exertional retrosternal chest pain for the past five days. The patient denied any fever, chills, or night sweats. Upon further questioning, the patient revealed that he was an ex-farm worker who worked in sugar cane fields where he was exposed to smoke daily for approximately 30-35 years. As per a family member, there has been no recent weight loss or hemoptysis noted. Prior to presentation, the patient was evaluated by his primary care physician and was prescribed a course of azithromycin without relief of his symptoms. Chest radiography revealed possible left lower lobe pneumonia and the patient was referred for admission to the hospital for the administration of intravenous antibiotics.

On presentation at the hospital, initial vital signs were a blood pressure of 141/79 mmHg, a heart rate of 112 beats per minute, an oxygen saturation of 96% on room air, a respiratory rate of 14 breaths per minute, and a temperature of 37.6 degrees Celsius. On physical examination, the patient seemed to be fatigued with the presence of bibasilar rhonchi on auscultation. The rest of the physical exam was unremarkable. Initial lab results are shown in Table 1.

**Table 1:** Laboratory results

Investigation	Results (normal values)
Total Bilirubin	2 (0.0-1.2mg/dl)
Direct Bilirubin	1.8 (0.0-0.3mg/dl)
Alkaline Phosphatase	230 (40-130U/L)
Albumin	2.2 (3.5-5.2 g/dl)
Aspartate Amino Transferase/Serum Glutamic-Oxaloacetic Transaminase (AST/SGOT)	25 (5-41U/L)
Alanine Aminotransferase/ Serum Glutamate-Pyruvate Transaminase (ALT/SGPT)	17 (5-40U/L)
Sodium Level	141 (136-145mmol/l)
Potassium Level	4.5 (3.5-5.1mmol/l)
Carbon Dioxide Level	19 (22-29mmol/l)
Chloride Level	106 (98-107mmol/l)
Blood Urea Nitrogen	42 (8-23mg/dl)
Creatinine	1.1 (0.70-1.20mg/dl)
Calcium Level	10.1 (8.6-10.4mg/dl)
Random Glucose	127 (74-99mg/dl)
Total Creatine Kinase	14 (20-200U/L)
Troponin I	<0.017 (0.010-0.030ng/ml)
White Blood Count	24.5 (4.80-10.80K/uL)
Hematocrit	34.1 (40-50%)
Hemoglobin	10.5 (13.3-17.7g/dl)
Platelet Count	74 (150-400K/uL)
Mean Corpuscular Volume	92.4 (80-100fl)
Prothrombin Time International Ratio	1.17 (0.87-1.13)
Activated Partial Thromboplastin time	26.6 (25-35 second)
Prothrombin Time	13.4 (10-13 second)

**Figure 1:**
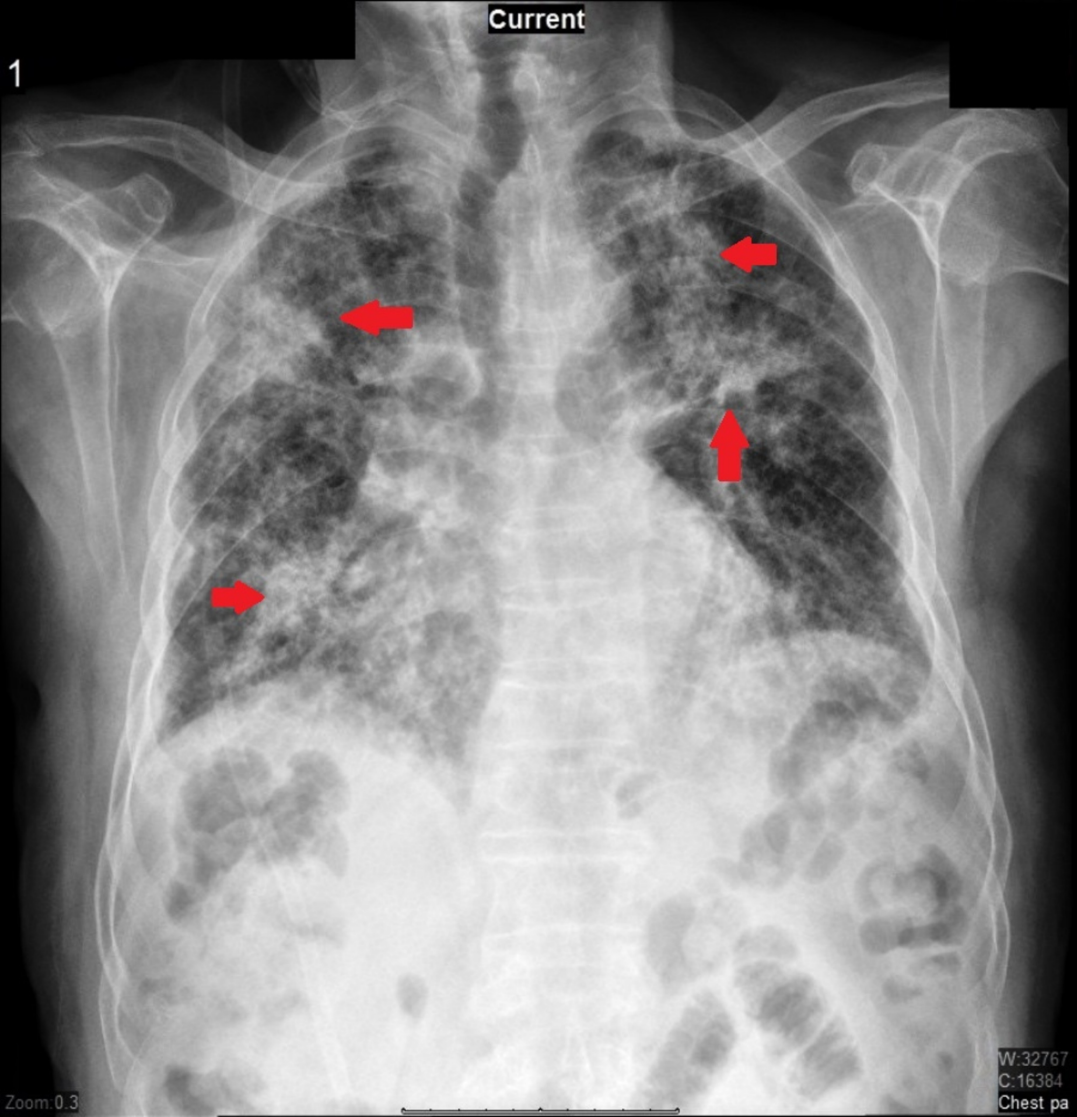
Chest radiography showing increased reticular markings and areas of consolidation suggestive of multilobular pneumonia

**Figure 2:**
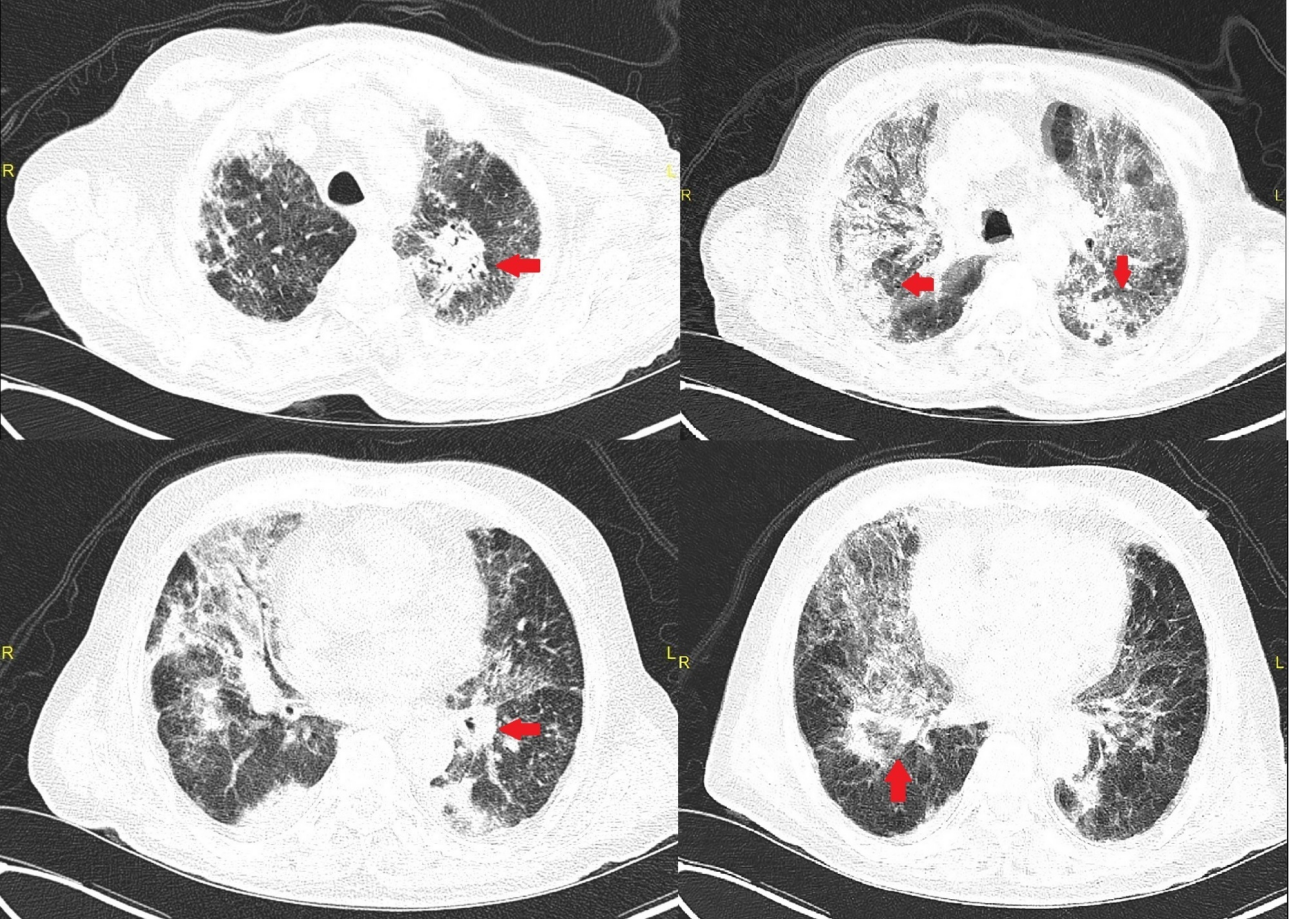
Computed tomography of the chest showing diffuse bilateral scattered patchy and nodular opacities throughout the lungs, possibly representative of multifocal pneumonia throughout the lungs Arrows point to consolidation with a pneumonia-like pattern

The patient was admitted and intravenous ceftriaxone and azithromycin were initiated, with a working diagnosis of community-acquired pneumonia. Approximately 36 hours into hospitalization, the patient became dyspneic and acutely hypoxic with oxygen saturation dropping to 80% on room air and the patient was started on high flow oxygen via nasal cannula (HFNC). A repeat chest x-ray showed multi-lobular pneumonia (Figure 1). Subsequent computed tomography (CT) of the chest showed diffuse bilateral scattered patchy and nodular opacities, with underlying scarring and fibrosis consistent with interstitial lung disease (Figure 2), according to radio-clinical criteria.

Despite high flow oxygen, the patient’s condition continued to decline, becoming more hypoxic. HFNC was changed to bi-level positive airway pressure, as the patient remained hypoxic on HFNC. Additional testing, such as respiratory viral panel, mycoplasma, streptococcus pneumonia, and HIV were all negative. Gram staining of the sputum culture was also found to be negative. Bronchoscopy was declined by family members due to the invasive nature of the procedure. Eventually, the sputum culture for acid-fast bacilli (AFB) returned to be positive. The patient was expectantly started on rifampin, isoniazid, pyrazinamide, and ethambutol (RIPE) therapy.

Despite targeted therapy, the patient further deteriorated, requiring intubation and was transferred to the intensive care unit due to hypoxic respiratory failure. The patient was started and maintained on RIPE therapy due to the AFB positive sputum culture. The patient continued to remain hypoxic despite the RIPE therapy and expired six weeks after admission. It was thought that the combination of interstitial process combined with the overlapping mycobacterial infection caused the demise of this patient.

## Discussion

We highlight a case of an 84-year-old male with underlying interstitial lung disease, which was further complicated by an active MTB infection. The diagnosis and management of active tuberculosis infections in patients with an underlying interstitial process may prove challenging, which demonstrates the uniqueness of this case. Very limited data are available exploring the relationship of MTB in ILD patients. In a study by Chung MJ et al., 143 consecutive patients with IPF were analyzed and it was found that the incidence of tuberculosis in patients with IPF was more than five times higher than that of the general population [3]. In addition, the most common CT findings in the study were subpleural nodules, lobar and segmental consolidations that are atypical manifestations for MTB, as they mimic lung cancer or bacterial pneumonia [3]. In our case, CT was described as diffuse, bilateral, scattered, patchy, and nodular opacities with confluence in the left apex measuring approximately 3.7 cm, possibly representing multifocal pneumonia. Due to such a pattern on radiological exams, wide-spectrum antimicrobial therapy was warranted. Further complicating our case was the absence of the typical presentation of an active MTB infection.

In an earlier study examining the relationship of chronic ILD and tuberculosis, Shachor et al. retrospectively reviewed 162 cases from 1970 to 1984 and found that 5%-6.2 % patients with chronic ILD have a positive culture for MTB, which was found to be approximately 4.5 times the incidence in the general population [4]. This indicates that the MTB incidence is higher in the ILD patients. From the literature, it seems apparent that the incidence of MTB in the ILD population is more than what is normally seen in the general population. However, not much data is available for other mycobacterial infections, which may also demonstrate a deleterious effect in the setting of ILD. In a 2012 study by Park et al. of 795 IPF patients, pulmonary infections with Mycobacterium tuberculosis (MTB) and non-tuberculous mycobacterium (NTM) were found in 35 (4.4%) and 16 patients (2.0%), respectively, which was higher than the general population. In addition, as expected, TB was more common in patients who were treated with immunosuppressants and among the 51 IPF patients who had mycobacterial infections, nine (18%) died during follow-up, three due to the progression of pulmonary tuberculosis [5].

Our patient proved to be a clinical challenge due to radiological findings that were consistent with changes seen in pneumonia. However, with a history of being an Ecuadorian farm worker who presented with pleuritic chest pain, hypoxia, and shortness of breath, ILD secondary to occupational hazards was also part of the differential. Appropriate measures were taken in order to treat the ILD without any improvement in the clinical picture. During the rapid deterioration of our patient, the eventual culture of the sputum was found to be positive for MTB. Despite the initiation of RIPE therapy alongside treatment measures for the ILD, the patient expired. The decision to culture the sputum was a fortuitous one, as the patient did not present with the traditional features of tuberculosis such as a cough, fever, chills, night sweats, weight loss, or hemoptysis. In fact, it has been observed that up to a third of MTB patients present with atypical symptoms (lack of greater than weeks of cough or fever) [6]. A further exacerbation of the situation could be attributed to the fact that older patients (>65 years old), as well as those who do not present without respiratory symptoms, are misdiagnosed more frequently, delaying the correct diagnosis of pulmonary tuberculosis [7]. The progression of ILD is acknowledged to be heterogeneous, with some patients remaining stable for prolonged periods, others showing more rapid steady progression, and still others succumbing to acute exacerbation [8]. It is quite possible that an acute exacerbation of the ILD was the onus for the reactivation of his latent tuberculosis or vice versa. The specific interaction between ILD and tuberculosis is still unknown, however, it has been suggested that a diffusely damaged lung parenchyma increases the susceptibility to dormant tuberculosis [4]. It is generally understood that interferon (IFN)-gamma and tumor necrosis factor (TNF)-alpha are the main players in preventing the reactivation of tuberculosis. However, a study in Japan showed that levels of TNF-alpha and IFN-gamma were significantly higher in ILD patients versus non-ILD patients [9]. It is plausible that the acute and chronic exacerbations of parenchymal damage of the lungs secondary to the ILD somehow inhibit the sustainment of granulomas via a non-cytokine pathway, thus allowing the mycobacterium to reactivate and spread. This phenomenon may have contributed to the rapid decline of our patient and his ultimate demise.

ILD with concomitant tuberculosis is a relatively rare occurrence, which may be challenging to diagnose. The symptoms of the ILD can often mask an underlying pulmonary infection and can result in a misdiagnosis of the patient. Due to the rarity of having these two illnesses at the same time, there are no established management protocols or algorithms. Current guidelines recommend the initial test in all cases of suspected ILD to include a urine dipstick, full differential blood cell count, serum urea, electrolytes and creatinine, and liver function tests. Other tests, such as tuberculin tests, are largely dependent upon clinical context [10]. However, a high clinical suspicion and, arguably, cases such as ours will demonstrate the importance of ruling out an MTB infection as a standard for all ILD patients, especially in the indigenous population.

## Conclusions

Herein we presented an interesting case of ILD with concomitant tuberculosis, a rare phenomenon which may present as a clinical challenge. As evidence suggests there is an increased presence of tuberculosis in the setting of ILD. A diagnostic approach inclusive of ruling out mycobacterium infection should be adopted. Clinicians involved in the management of ILD should take consideration of possible concomitant infection.

## References

[1] World Health Organization (2017). Global Tuberculosis Report 2017. http://apps.who.int/iris/bitstream/10665/259366/1/9789241565516-eng.pdf?ua=1.

[2] Kaplan MH, Armstrong D, Rosen P (1973). Tuberculosis complicating neoplastic disease. A review of 201 cases. Cancer.

[3] Chung MJ, Goo JM, Im JG (2004). Pulmonary tuberculosis in patients with idiopathic pulmonary fibrosis. Eur J Radiol.

[4] Shachor Y, Schindler D, Siegal A, Lieberman D, Mikulski Y, Bruderman I (1983). Increased incidence of pulmonary tuberculosis in chronic interstitial lung disease. Thorax.

[5] Park SW, Song JW, Shim TS (2012). Mycobacterial pulmonary infections in patients with idiopathic pulmonary fibrosis. J Korean Med Sci.

[6] Miller LG, Asch SM, Yu EI, Knowles L, Gelberg L, Davidson P (2000). A population-based survey of tuberculosis symptoms: how atypical are atypical presentations?. Clin Infect Dis.

[7] Mathur P, Sacks L, Auten G, Sall R, Levy C, Gordin F (1994). Delayed diagnosis of pulmonary tuberculosis in city hospitals. Arch Intern Med.

[8] Travis WD, Costabel U, Hansell DM (2013). An official American Thoracic Society/European Respiratory Society statement: update of the international multidisciplinary classification of the idiopathic interstitial pneumonias. Am J Respir Crit Care Med.

[9] Gono T, Kaneko H, Kawaguchi Y (2014). Cytokine profiles in polymyositis and dermatomyositis complicated by rapidly progressive or chronic interstitial lung disease. Rheumatology (Oxford).

[10] Bradley B, Branley HM, Egan JJ (2008). Interstitial lung disease guideline: the British Thoracic Society in collaboration with the Thoracic Society of Australia and New Zealand and the Irish Thoracic Society. Thorax.

